# *CryoSift*: an accessible and automated CNN-driven tool for cryo-EM 2D class selection

**DOI:** 10.1107/S2053230X25008866

**Published:** 2025-11-07

**Authors:** Jan-Hannes Schäfer, Austin Calza, Keegan Hom, Puneeth Damodar, Ruizhi Peng, Nebojša Bogdanović, Gabriel C. Lander, Scott M. Stagg, Michael A. Cianfrocco

**Affiliations:** a Department of Integrative Structural and Computational Biology Scripps Research La Jolla California USA; b Institute of Molecular Biophysics Florida State University Tallahassee Florida USA; c Department of Biological Sciences Florida State University Tallahassee Florida USA; d Department of Biological Chemistry, Life Sciences Institute University of Michigan-Ann Arbor Ann Arbor Michigan USA; University of Leeds, United Kingdom

**Keywords:** automation, data processing, cryo-EM, single-particle cryo-EM, deep learning

## Abstract

Single-particle cryo-electron microscopy (cryo-EM) has become an essential tool in structural biology. However, automating repetitive tasks remains an ongoing challenge in cryo-EM data-set processing. Here, we present a platform-independent convolutional neural network (CNN) tool for assessing the quality of 2D averages to enable the automatic selection of suitable particles for high-resolution reconstructions, termed *CryoSift*. We integrate *CryoSift* into a fully automated processing pipeline using the existing *cryosparc-tools* library. Our integrated and customizable 2D assessment workflow enables high-throughput processing that accommodates experienced to novice cryo-EM users.

## Abstract 2

*CryoSift* is a CNN-based tool that assesses the quality of 2D class averages. The incorporation of *CryoSift* into *cryoSPARC* workflows enables automated particle-stack cleaning.

## 1. Introduction

Single-particle cryogenic electron microscopy (cryo-EM) has rapidly grown from a niche method into a fundamental tool in structural biology (Kühlbrandt, 2014 ▸). While this evolution has made the processing of challenging macromolecules more accessible, the cryo-EM community would benefit from reliable automated processing workflows. Current single-particle analysis (SPA) applications in cryo-EM are required to be reliable, fast and user-friendly. The widespread adoption of cryo-EM has been facilitated by graphical user interfaces. Thus, new automated pipelines must not only be accessible via the command line but also incorporated into user-friendly interfaces.

Many steps in preprocessing raw cryo-EM data to particle stacks and 2D class averages are nearly automated. The stack of sequential frames that comprise a single cryo-EM micrograph must first be aligned to account for electron beam-induced motions during acquisition and the aligned frames then subsequently averaged so that the aberrations associated with the contrast transfer function (CTF) can be estimated. These processing steps are performed on each of the thousands of exposures that are associated with a given single-particle cryo-EM data set. Live and streamlined pre-processing tools have been implemented in popular processing environments such as *cryoSPARC* (Punjani *et al.*, 2017 ▸) and *RELION* (Kimanius *et al.*, 2021 ▸), allowing users to adjust data collection according to their specifications. However, the subsequent detection, extraction and classification of particles remains an iterative process that typically requires user intervention.

Since cryo-EM produces high-resolution reconstructions from noisy, heterogeneous particle collections, classifying individual 2D projection of particles into homogeneous groups is often nontrivial. Automated workflows help address two different bottlenecks. Firstly, there are new users who need guidance at the decision points, such as deciding what are ‘good’ and ‘bad’ images or class averages. Tools that provide assessment metrics are useful for guiding such users to make good decisions for their structure determination, overcoming a ‘user-input bias’. Secondly, for well established samples where investigators are looking at variations, cofactors, drugs bound and more, it is useful to have tools that take the manual decision-making steps out of the workflow so that multiple samples can be processed systematically, as shown for implementing routine processing of G protein-coupled receptors (Danev *et al.*, 2021 ▸).

Substantial progress has been made towards a more user-friendly and unsupervised selection of suitable 2D projections. *RELION*’s *Class Ranker* automates 2D class selection using a trained convolutional neural network (CNN) that labels each class and applies a cutoff, effectively ranking and selecting suitable classes for downstream analysis (Kimanius *et al.*, 2021 ▸). Similarly, *SPHIRE* (Moriya *et al.*, 2017 ▸) has implemented a similar CNN-based labeling approach termed *Cinderella* (https://github.com/MPI-Dortmund/sphire_classes_autoselect), which separates 2D classes into good and bad class averages. *Cinderella* offers both a pre-trained CNN model and training options for user data sets. While these methods utilize CNNs, *cryoSPARC* offers its own approach for automating 2D class selection through similarity comparisons to projections of a user-provided 3D volume. This approach, currently under active development, is expected to be most valuable for processing pipelines of known targets, using shape similarity and pixel-by-pixel similarity to calculate projections of the provided 3D volume. Our own previous work on removing subjective decision-making for 2D class selection as part of a progressively user-free processing pipeline (Li *et al.*, 2020 ▸) also relies on a CNN-based classifier that automatically selects suitable 2D class averages. This classifier (2*DAssess*) was trained on a labeled data set of 2D class averages, preprocessed and categorized into four classes: good, clip, edge and noise. While 2*DAssess* showed promise, it was command-line only and was not incorporated into any user-friendly workflows, limiting its adoption.

Current approaches for automated 2D class selection are either platform-dependent (for example *RELIONClass Ranker*) or standalone command-line tools that do not readily support cross-platform integration (for example 2*DAssess*). In this study, we addressed this limitation by developing *CryoSift*, a 2D class-average quality assessor that is platform agnostic (*i.e.* able to assess data from *RELION* or *cryoSPARC*) to enable unsupervised data-set processing. We show the utility of this tool by incorporating it into *cryoSPARC* using the *cryosparc-tools* API, providing customizability for advanced users while maintaining ease of use for cryo-EM beginners utilizing the default *cryoSPARC* implementation.

## 2. Materials and methods

### 2.1. Training of the 2D assessor *CryoSift*

#### 2.1.1. Generating 2D averages for training

EMPIAR data sets from Tables 1 ▸ and 2 ▸ were imported into *cryoSPARC* (v.4.1.6) and preprocessed with *CTFFIND*4 and patch-based motion correction for imported movies. Templates for template picking were created from the associated EMDB maps (Tables 1 ▸ and 2 ▸) using the *cryoSPARC* ‘Create Templates’ job. Particle dimensions from measurements in *ChimeraX* (Pettersen *et al.*, 2021 ▸) were used in template picking. Picked particles were extracted using box sizes about 1.5-fold the particle diameter and binned by two. The extracted particles were subjected to two or three rounds of 2D classification. To cover a broad range of 2D class qualities, the projections from all iterations were used for labeling. Additionally, pre-labeled 2D averages from *RELION*’s 2D *Class Ranker* (EMPIAR-10812) were utilized. The class averages and selected metadata, including the pixel size, resolution estimates from Fourier ring correlation (FRC) and relative class distribution, were extracted from the processing runs and were presented to the CNN for training.

#### 2.1.2. Generating mass metadata for CNN training and assessment

Masses for each class average were estimated based on the pixel intensities and pixel sizes. The sum of the pixel intensity of the particle signal in class averages was calculated as follows. (i) Define all pixels three standard deviations above the mean as the particle signal and the remainder as background. (ii) Subtract the mean of the background from the mean of the particle. (iii) Sum the intensity values for the particle pixels and multiply by the pixel size squared. A calibration curve for converting the summed intensities to masses was determined by taking EMPIAR data of homogeneous particles (Tables 1 ▸ and 2 ▸, Supplementary Fig. S1) with known masses and plotting the pixel intensities versus their known masses. This resulted in a linear plot, and the slope was used to determine a calibration factor that was multiplied by the summed intensity values for each class average to convert them into kDa masses. A plot of the particles with known masses over the estimated masses for their class averages is shown in Supplementary Fig. S1. The mass information was incorporated into the CNN by including the deviations from the mean, median and mode mass as part of the metadata used during training and evaluation.

#### 2.1.3. Expert labeling of 2D averages for CNN training

**Figure 1:**
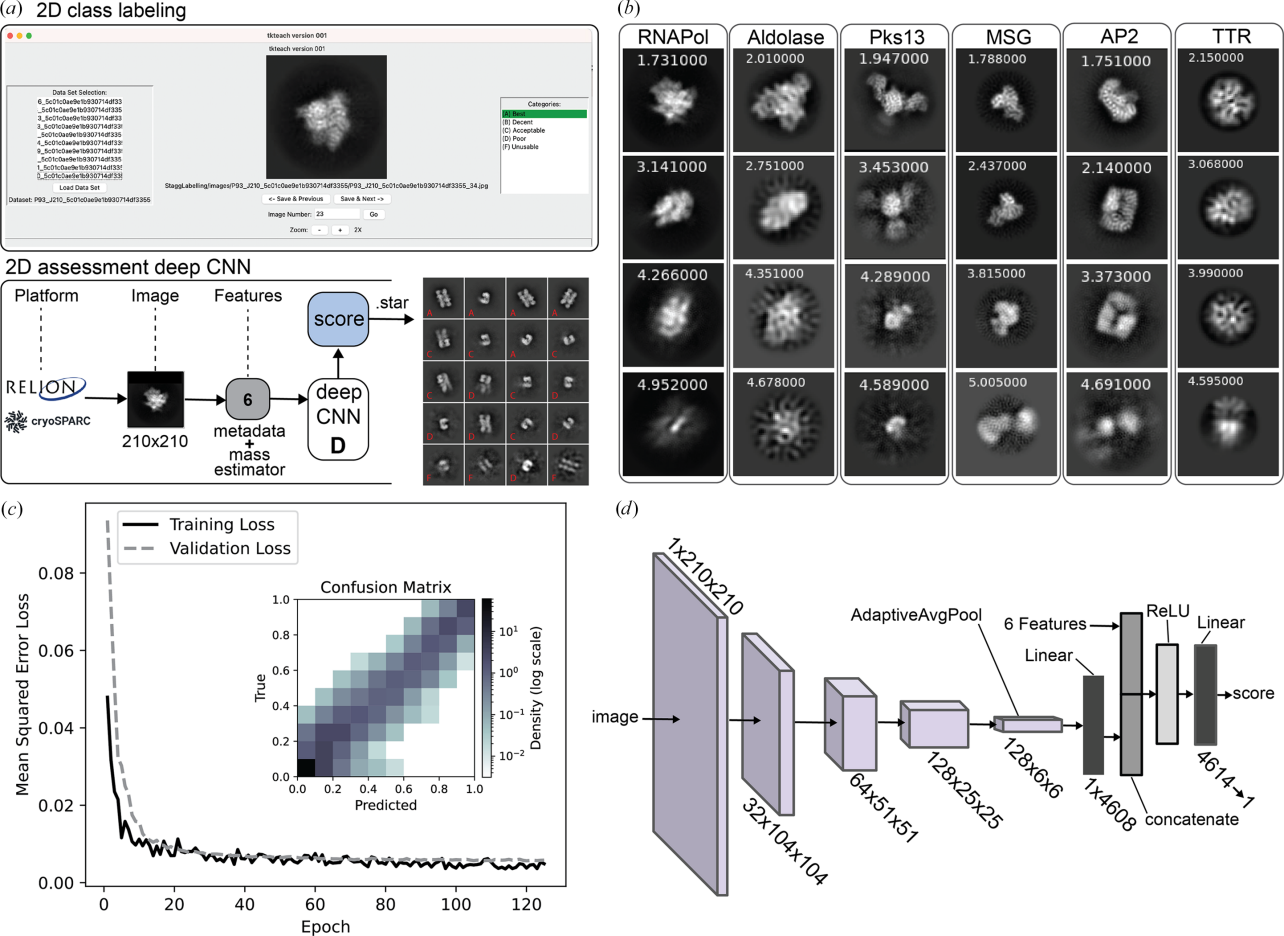
Architecture, training and benchmarking of *CryoSift*. (*a*) GUI of *tkteach* for 2D class labeling and basic architecture of the deep convolutional neural network of *CryoSift*. *RELION* or *cryoSPARC* 2D projections of 31 × 31 to 210 × 210 px input. Three data features from the mass estimator and three metadata features (FRC resolution, class distribution and pixel size) are also fed into the model, resulting in a predicted quality score. Example output of AP2 averages with grade-based labels (red). (*b*) 2D averages with predicted quality scores are grouped by protein, sampled across the full range of class-quality scores. (*c*) Mean-square error loss over epochs of training and validation with features. The inset shows the prediction error between true and predicted score as a confusion matrix (density on a log scale). (*d*) Overview of the CNN layers (details are given in Supplementary Fig. S2).

Manual labeling of 2D class averages for training was implemented using the Python-based image-categorization tool *tkteach* (https://github.com/rmones/tkteach). 2D class averages from *cryoSPARC* were extracted, converted to JPGs and used as input for an adopted version of *tkteach*. The *tkteach* GUI allows the easy labeling of the classes (Fig. 1 ▸*a*) and storage of the associated labels for training of the CNN. Researchers from the Stagg, Cianfrocco and Lander laboratories were involved in labeling the 2D class images. Assessors were provided a rubric for manually grading class averages as follows: A, best, secondary structure, very sharp looking; B, decent, secondary structure, some fuzzy regions; C, acceptable, overall shape of a particle, some domain details, little to no secondary structure, D, poor, particle-like shape but fuzzy with artifacts; F, unusable, nothing resembling a particle, artifacts in the background. Assessors were provided with example labeled averages to aid in making consistent evaluations (Supplementary Fig. S2). The pooled averages were used for training the CNN.

### 2.2. Designing and training *CryoSift*

Our 2D class-average assessor *CryoSift* was built using residual connections as described in the original ResNet paper (He *et al.*, 2015 ▸), which allows outputs from earlier layers to bypass one or more subsequent layers, helping to preserve gradient flow during backpropagation and mitigating the vanishing gradient problem caused by repeated application of the chain rule. *CryoSift* applies two consecutive convolutional filters, each with a 2 × 2 filter and a stride of 2, instead of pooling. This approach achieves downsampling while also enabling the network to learn weighted combinations of features at each stage, effectively functioning as a form of weighted average pooling. The CNN also employs batch normalization, which takes an input tensor and tries to normalize it to a mean of 0 and a standard deviation of 1, averaged across all observed images in the training set. Finally, the CNN employs adaptive average pooling to allows input images of any size greater than 31 × 31. This was enabled through the use of PyTorch’s *AdaptiveAvgPool*2*D*, which is a pooling layer that adaptively chooses pooling size and stride according to the image’s size, such that the output size of the pooling layer is always the same, in this case 6 × 6.

*CryoSift* was trained using Python 3 and PyTorch. The Adam optimizer (Kingma & Ba, 2017 ▸) was used for model generalization and convergence. The learning rate was 0.0001 with a weight decay of 0.0001. The batch size was 32, and the model was trained for 200 epochs with little change to validation loss after approximately 60 epochs.

### 2.3. Testing and validation of *CryoSift*

For testing *CryoSift*, ten single-particle cryo-EM data sets were downloaded from the EMPIAR repository, covering a wide range of molecular weights. These include aldolase, DNA protection during starvation protein (DPS), streptavidin, 70S ribosome in complex with VmlR2, P-Rex1-G-beta-gamma signaling scaffold (P-Rex1G), RNA polymerase sigma N (RNAPol), malate synthase G (MSG), mycobacterial polyketide synthase 13 (Pks13), mitochondrial respiratory complex I (Complex-I) and an in-house data set of mouse apoferritin. The associated identifiers and data-collection parameters are listed in Table 3 ▸.

### 2.4. Sample preparation and cryo-EM of mouse apoferritin

Mouse apoferritin (ApoF; heavy chain) was prepared following our published protocol (Basanta *et al.*, 2022 ▸), yielding a concentrated sample of 15 mg ml^−1^ in 30 m*M* HEPES pH 7.5, 150 m*M* NaCl, 1 m*M* DTT with 5%(*v*/*v*) glycerol. Graphene grids were prepared following our established protocol (Basanta *et al.*, 2023 ▸). In brief, methylmethacrylate-supported graphene was transferred onto Quantifoil UltrAuFoil 0.6/1.0 400 mesh grids and treated with ozone to render the graphene hydrophilic. Apoferritin was diluted to 1.5 mg ml^−1^ in glycerol-free buffer and applied to the grids, which were plunge-frozen manually at 4°C at 90% humidity for 3 s. 900 movies were collected on a 200 keV Arctica (Thermo Fisher) equipped with a Falcon 4 (Thermo Fisher) at a total electron exposure of 40 e^−^ Å^−2^, a nominal magnification of 190 000 and an uncalibrated pixel size of 0.74 Å per pixel. Movies were collected automatically using *EPU* (v.3.9).

### 2.5. Standardized processing of single-particle data sets in *cryoSPARC*

All data sets in Table 3 ▸ were processed in *cryoSPARC* (v.4.6), following a standardized processing scheme. If required, beam-induced motion in the imported EMPIAR data sets was corrected using patch-motion correction, followed by CTF estimation with *Patch-CTF*. For the in-house ApoF data set, micrographs were curated using a CTF cutoff at 4 Å and an astigmatism of lower than 600 Å, yielding a stack of 277 micrographs. Picking templates were generated from a small stack of 50 micrographs using a blob picker with the diameters in Table 3 ▸. The subsequently template-picked particles were extracted in a box matching twice the particle diameter, Fourier-cropped to 100 pixels (px). The resulting 2D classification was used as input for the automated processing pipeline using our *cryoSPARC* tools implementation with mask sizes 20% larger than the particle diameter from Table 3 ▸. Nondefault parameters were automatically selected from the user-provided extraction box. Box sizes above 300 px are considered large, while boxes below 200 px are considered small. All in-between sizes use the default settings. All particles were classified using a 100 px Fourier-cropped box, allowing adaptive binning for larger boxes. Small inputs are processed with the new 2D Classification (Small Particle) job type, using 3 Å maximum resolution and initial class uncertainty factor 3, turned Force max over poses/shifts off, over 40 online-EM iterations with a Batchsize of 400. *Ab initio* reconstructions were generated using a maximum resolution set to 3 Å, an initial resolution of 35 Å (default), initial minibatch size 300 and final minibatch size 1000. Re-processing of the Pks13, DPS and Complex-I data sets in *RELION*5 was performed using pre-processed particle stacks from *cryo­SPARC*. For file conversion and transfer to *RELION*5, *pyem* (https://github.com/asarnow/pyem/) was utilized. 2D classification in *RELION*5 was performed with *VDAM* enabled, *T* = 2 and 20 classes.

## 3. Results

### 3.1. Design of the CNN-based 2D assessor *CryoSift*

Building on *RELION*’s *Class Ranker* (Kimanius *et al.*, 2021 ▸) and our previous 2*DAssess* tool (Li *et al.*, 2020 ▸), we developed a 2D assessment tool that employs a deep CNN to evaluate 2D class averages and their platform-independent metadata, termed *CryoSift*. The model incorporates pixel size, resolution estimates, relative class distribution, deviation from mean mass, deviation from median mass and deviation from mode mass as input features (Fig. 1 ▸*a*). Images with higher class distribution, better resolution and smaller pixel size typically produce sharper, higher-quality projections, enabling the CNN to leverage these parameters for improved classification. Based on the provided images and associated metadata, the trained CNN assigns continuous quality scores ranging from 1.0 (best) to 5.0 (worst). The model was trained using a mean-square-error (MSE) loss function, not a Softmax-based function (which would limit the score to a certain range). This means that the model is technically able to predict real numbers as the score, relative to its training-scores range. This results in open score boundaries, and for cases of classes worse than the worst training class scores higher than 5 are given. For classes better than the best training class, scores smaller than 1 are assigned. The 2D class evaluator accepts images from both the *RELION* and *cryoSPARC* platforms, enabling platform-independent data processing with cross-platform compatibility.

Our algorithm automatically Fourier-scales all input images to 210 × 210 pixels to allow adaptive feature extraction. Images smaller than these dimensions are zero-padded to 210 pixels, while larger images are down-sampled. This approach preserves native image features to enhance prediction accuracy, with a minimum input size requirement of 31 × 31 pixels for zero-padding operations. Padding each 2D class image to a fixed size instead of allowing variable inputs during training results in two main advantages. (i) Mini-batch training: the model updates its weights after seeing a batch of images (for example 32), instead of after every image. This allows a much smoother weight convergence, plus it leverages GPU parallel hardware to speed up training. (ii) Batch normalization: this is a common neural network method that greatly speeds up training convergence. Batch normalization requires the model to train on mini-batches of data.

The architecture of our deep CNN is detailed in Fig. 1 ▸(*d*) (layer details in Supplementary Fig. S3). The CNN was trained on 32 204 total 2D averages: 26 389 pre-labeled images from EMPIAR-10812 (used in *RELION*’s *Class Ranker*) combined with 5815 2D class averages from *cryoSPARC* (Tables 1 ▸ and 2 ▸), labeled by members of the three participating laboratories. The combined data sets include 2D averages generated by both *RELION* and *cryoSPARC*, effectively training the CNN to recognize and account for platform-specific differences (Fig. 1 ▸*a*). Consequently, *CryoSift* can generalize its predictions across *RELION*- and *cryoSPARC*-generated images. The model outputs labels in STAR file format that users can inspect using the *relion_display* function (Fig. 1 ▸*b*).

*CryoSift* can be installed as a standalone command-line tool (https://github.com/sstagg/Magellon/tree/main/Sandbox/particle_processor) and incorporated into *cryoSPARC* user workflows. Alternatively, users can access the online version of the assessor at https://www.cryosift.org (Supplementary Fig. S4) and can upload either *RELION*- or *cryoSPARC*-generated 2D class averages to inspect their class quality and optionally contribute their own class labels to refine the CNN model. The latter feature will allow us to continually refine the model with different types of samples and conditions. In this way, the model has the potential to become more accurate and generalizable over time.

Overall, our redesigned 2D class ranker *CryoSift* offers several advances over our earlier 2*DAssess* tool. 2*DAssess* offered no GUI support, while *CryoSift* offers an easy-to-use web server. Its improved CNN architecture supports adaptive average pooling, which is not included in either *RELION*’s *Class Ranker* or 2*DAssess*, excluding variable image size as input. The fixed four-category labeling of 2*DAssess* cannot pick up on minor differences in 2D class quality, which the continuous labeling within *CryoSift* can. Adopting *RELION*’s use of metadata contributed to variations in the final class scoring, adding to *CryoSift*’s overall improved accuracy.

### 3.2. Automation with *cryosparc-tools*

We integrated output from *CryoSift* as a filter for automatic selection of 2D averages using the *cryosparc-tools* Python library (https://github.com/cryoem-uoft/cryosparc-tools). Iterative 2D classification is helpful to remove ‘bad’ particles from a given data set. For instance, even well aligning 2D classes with visible secondary-structure features retain heterogeneity. Moreover, iterative 2D classification also helps to mitigate the ‘attractor effect’, where less frequent views or low signal-to-noise ratio images may be attracted together (Chung *et al.*, 2020 ▸). Interfacing with *cryoSPARC* enables automated and standardized selection of 2D classes through iterative selection and classification of well aligning classes, while noise and poorly aligning particles are discarded. User-defined thresholds guide the sorting of good and bad particles.

**Figure 2:**
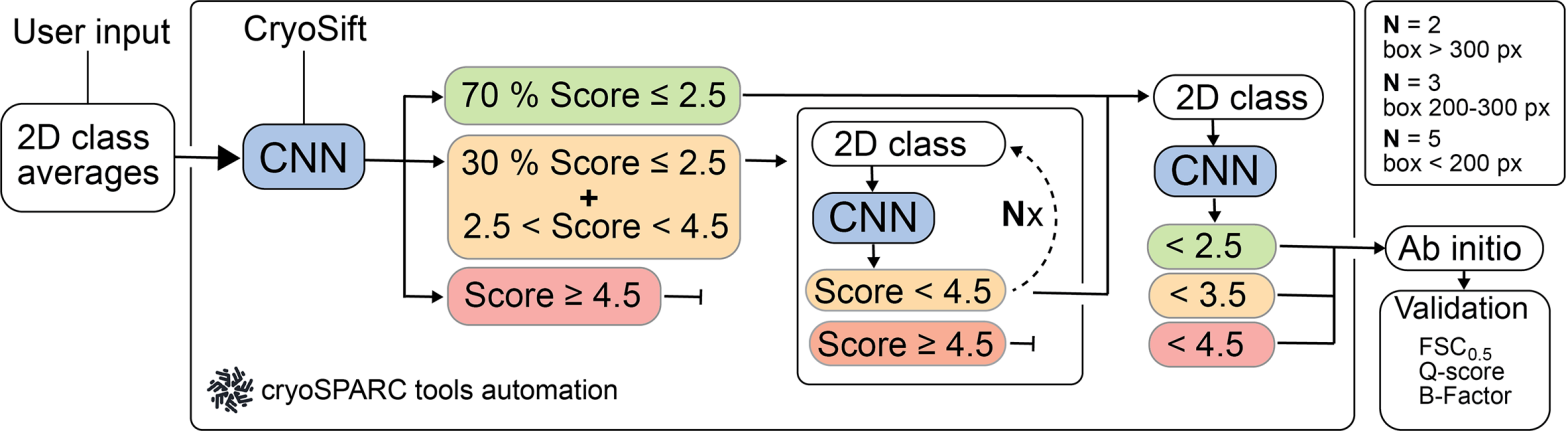
Automated workflow scheme for iterative 2D classification using *CryoSift* with *cryosparc-tools*. *CryoSift* generates quality labels from user-provided 2D projections and is forwarded to the *cryosparc-tools* API. A user-provided cutoff score defines the Select/Reject list for the 2D Select job in *cryoSPARC* and iterates through 2D classification, class-quality labeling and selection for *N* rounds: *N* = 2 for boxes >300 px, *N* = 3 for boxes between 200 and 300 px and *N* = 5 for boxes <200 px. All particles passing the cutoff criterion are pooled and subjected to an *ab initio* reconstruction. The reconstruction quality is assessed from the Fourier shell correlation (FSC) to a simulated prior (FSC cutoff at 0.5). The atom inclusion is calculated using a corresponding model file and reported as the mean overall *Q*-score.

Our *cryosparc-tools* implementation of *CryoSift* currently iterates 2D classification and labeling over particles with labeling scores between 2.5 and 4.5. In each iteration, 70% of particles with scores better than or equal to 2.5 are excluded from further iterations, while 30% of particles with scores better than or equal to 2.5 are accepted for the iterative classification. Particles with a *CryoSift* score worse than or equal to 4.5 are discarded. In the following iterations, particles with scores better than 4.5 are pooled, while particles with worse labels are rejected. The number of iterations of processing depends on the extraction box sizes as an indirect measure of particle size. Consequently, box sizes of <200 px iterate five times and particles with box sizes between 200 and 300 px iterate three times, while larger box sizes only iterate twice through the classification and labeling (Fig. 2 ▸). Subsequently, all particles passing the 4.5 cutoff are pooled and subjected to final 2D classification, then split into three batches with score thresholds of 2.5, 3.5 and 4.5. These batches undergo re-extraction without binning and are subjected to *ab initio* reconstruction. The integrated *CryoSift* framework is illustrated in Fig. 2 ▸.

### 3.3. Validation and testing of *CryoSift*

For validation of our CNN model, 3220 class averages, or 10% of the original data set, were used. This subset was excluded from training to maintain independence of validation. Our model was trained for 200 epochs in PyTorch using an Adam optimizer (Kingma & Ba, 2017 ▸) for the mean-squared error of the predicted score against the labeled score. A learning rate and weight decay of 0.0001 with batch sizes of 32 have been identified as optimal through trial and error, and achieved about 0.0055 MSE loss on the validation data, with insignificant improvement after 60 epochs (Fig. 1 ▸*c*). The mean-square error (MSE) between true (labeled) and predicted scores was calculated after conversion to a bin range from 0 to 1 to match the 2D *Class Ranker* labels from *RELION*5. An average MSE of 0.0039 was calculated. While high bins and low bins showed the least disagreement with the labels, higher discrepancy was observed for the mid-range scores (bins around 0.5; Fig. 1 ▸*c*).

**Figure 3:**
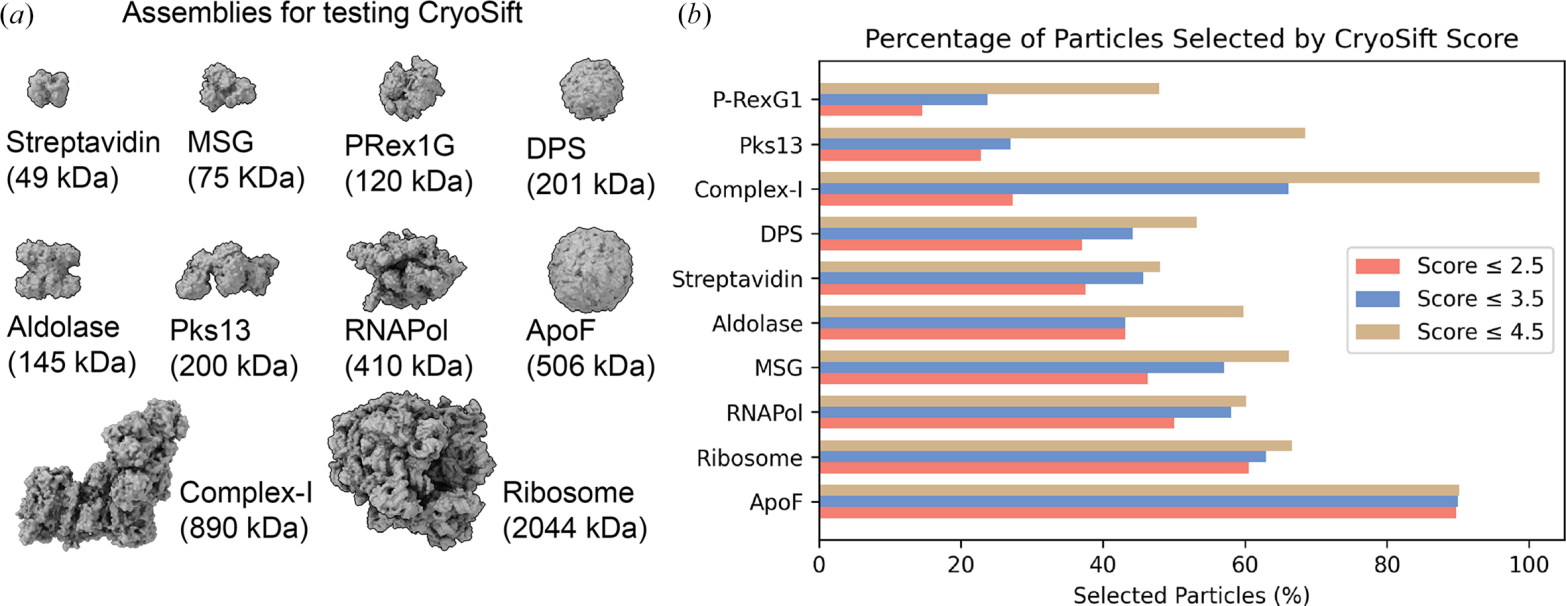
Particle statistics of the automated, unsupervised iterative 2D classification workflow. (*a*) Tested proteins or protein complexes, ordered by increasing molecular weight. (*b*) Fraction of selected particles according to *CryoSift* scores of ≤2.5 (coral), ≤3.5 (blue) and ≤4.5 (tan), ordered by fraction of selected particles.

To test *CryoSift*, ten data sets were selected, covering a broad range of molecular weights (Fig. 3 ▸*a*), oligomeric states and commonly used microscope setups (Table 3 ▸). After standardized user-based preprocessing (outlined in Section 2), all data sets were subjected to our unsupervised iterative 2D classification using the *cryosparc-tools* API as shown in Fig. 2 ▸. To validate the impact of particle selection based on their *CryoSift* score, we grouped the particles into three clusters with scores lower than or equal to 2.5, lower than or equal to 3.5 and lower than or equal to 4.5. Since refinements rely on particle-weighting schemes (Punjani *et al.*, 2020 ▸), we generated initial reconstructions using the ‘Ab-initio reconstruction’ job type in *cryoSPARC* without symmetry. Recognizing that using stochastic gradient descent with randomized initial seeds can generate biased volumes, we calculated a global resolution estimate of the *ab initio* reconstructions against simulated data. To minimize further diverging orientation bias from random-seed generation, we used cloned parameters, including the random seed, for each *ab initio* reconstruction job across all three *CryoSift* cutoff particle stacks. The simulated data were generated using the corresponding atomic models (Table 3 ▸), filtered to 8 Å and resampled to match the box and voxel size of the reference *ab initio* reconstruction using *ChimeraX*’s vop command. The FSC was calculated using *EMAN* (v.2.12; Tang *et al.*, 2007 ▸). Additionally, we calculated the *Q*-score as a measure of atom inclusion using the *ChimeraX* toolshed (https://github.com/tristanic/chimerax-qscore) and used *cryoSPARC*’s ResLog analysis job to calculate the *B* factor as an overall estimate for data quality and impact of the imaging conditions and chosen processing steps, focusing on the applied 2D accessor cutoffs (Stagg *et al.*, 2014 ▸).

The fraction of selected particles greatly varied across the chosen data sets (Fig. 3 ▸*b*). The accepted particles per iteration are shown in Supplementary Fig. S6. It should be noted that the fraction of accepted particles is affected by the quality of detecting particles and generally improves with increasing signal to noise (S/N). Additionally, larger particles (ribosomes) have fewer false-positive picks compared with smaller particles (streptavidin). In our processing workflow, particle picks were not subjected to particle inspection and curation to highlight the inherent false-positive picking in noisy data sets.

The mass-to-volume ratio impacts 2D class qualities, as shown by the fraction of accepted particles (Fig. 3 ▸*b*). ApoF on graphene shows the highest fraction of accepted particles, with minor differences between the *CryoSift* cutoff thresholds. A strong difference between accepted particles across the *CryoSift* score was present for the membrane protein Complex-I, potentially caused by detergent micelle 2D averages (Supplementary Fig. S6). For the PRex-1G data set, only about half of the particles are selected and only about a quarter of the particles pass the 3.5 *CryoSift* cutoff. For most data sets, the difference in accepted particles between the 4.5 and 3.5 *CryoSift* cutoffs is greater than the difference between the 3.5 and 2.5 cutoffs, indicating an optimum for retaining rare views while discarding poorly aligning particles. To validate the quality of the selected particles using our *CryoSift* score, we generated reconstructions for each of the *CryoSift* cutoffs. Since *cryoSPARC* integrates particle weighting in refinement jobs, we used the *ab initio* reconstruction job type without enforcing symmetry, as outlined in Section 2. All reconstructed volumes are ordered by increasing resolution and color-coded by *CryoSift* cutoff scores in Fig. 3 ▸(*e*).

**Figure 4:**
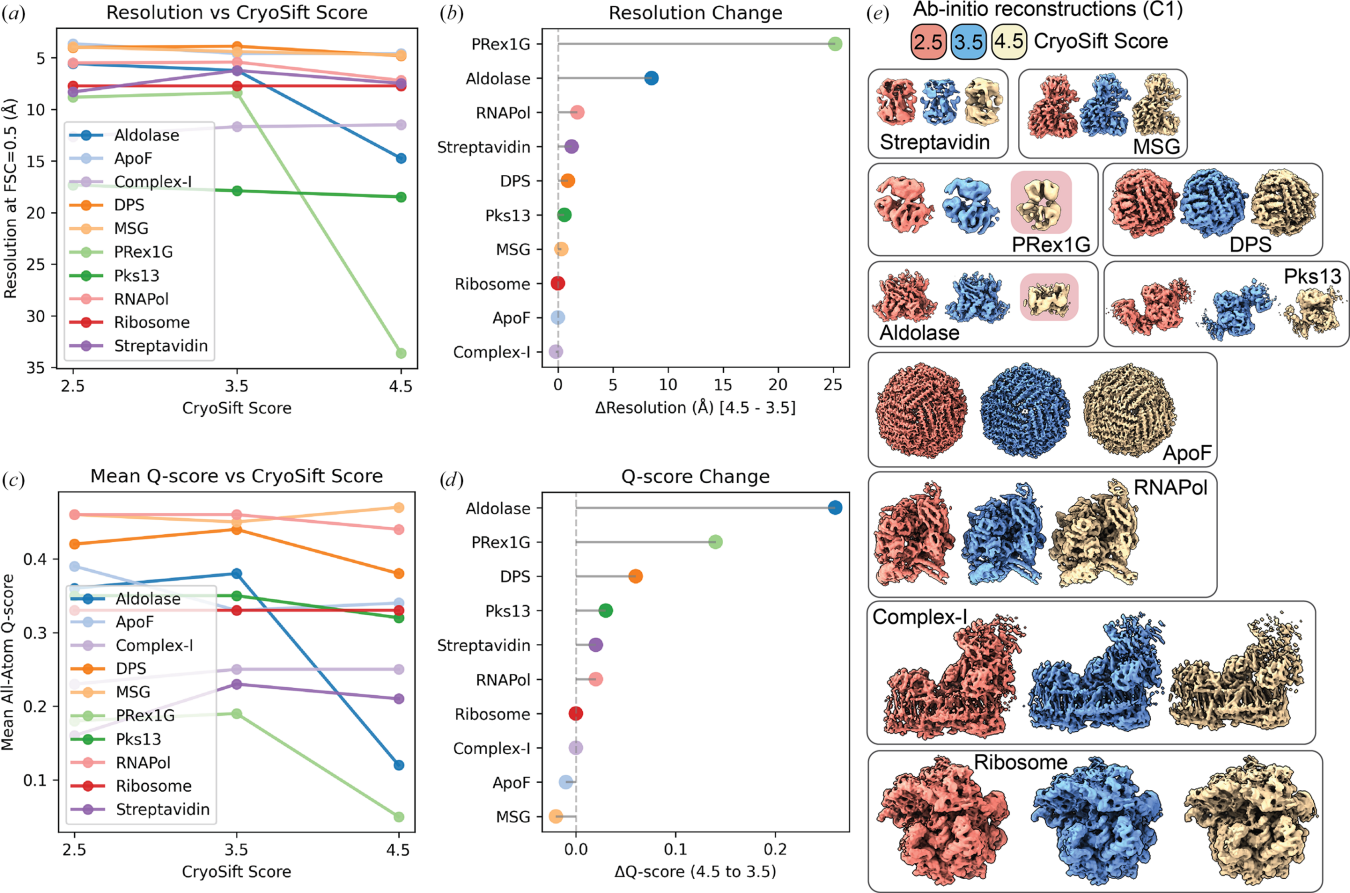
Validation of the reconstructions from the unsupervised iterative 2D classification workflow. (*a*) Resolution of *ab initio* reconstructions across three different *CryoSift* scores using the FSC_0.5_ against simulated volumes. (*b*) Resolution improvement from *CryoSift* score 4.5 to 3.5, showing the removal of unsuitable particles. (*c*) All-atom inclusion into *ab initio* reconstructions across three different *CryoSift* scores using the mean all-atom *Q*-score. (*d*) All-atom inclusion improvement from *CryoSift* score 4.5 to 3.5. (*e*) *Ab initio* reconstructions used for (*a*)–(*d*) color-coded by the *CryoSift* scores. *Ab initio* reconstructions were calculated without imposing symmetry (*C*1) and color-coded by their respective *CryoSift* score, 2.5 (coral), 3.5 (blue) and 4.5 (tan), and ordered by increasing molecular weight. Failed reconstructions for aldolase and PRex-1G are highlighted in red.

To validate the quality of the reconstructions, we calculated the FSC to simulated data using atomic coordinates from Table 3 ▸. Specifically, the FSC at threshold 0.5 was used to define the resolution of the reconstruction and is plotted against the selected *CryoSift* cutoffs in Fig. 4 ▸(*a*). Plotting the resolution differential between the 4.5 and 3.5 cutoff (Fig. 4 ▸*b*) indicates a negligible impact for several selected data sets, but a substantial resolution improvement for some data sets (for example aldolase and PRex-1G). Additionally, we used the *Q*-score as an indicator of map quality and interpretability. The mean all-atom *Q*-score over the *CryoSift* scores is shown in Fig. 4 ▸(*c*) and the *Q*-score change between cutoffs of 4.5 and 3.5 is visualized in Fig. 4 ▸(*d*). The map–model fit shows improvements for many data sets, but ApoF and MSG show a loss of map interpretability when applying a stricter 2D cutoff. Both selected validation criteria indicate that curating 2D classes can help improve the quality of 3D reconstructions. While global resolution estimation using the FSC offers an easy comparison to the ground truth, local map interpretability reported using the *Q*-score improves when removing low-quality particles, which might carry radiation damage or air–water interface denaturation, or have been collected from areas of low S/N on the grid (thick ice, non-vitreous ice).

**Figure 5:**
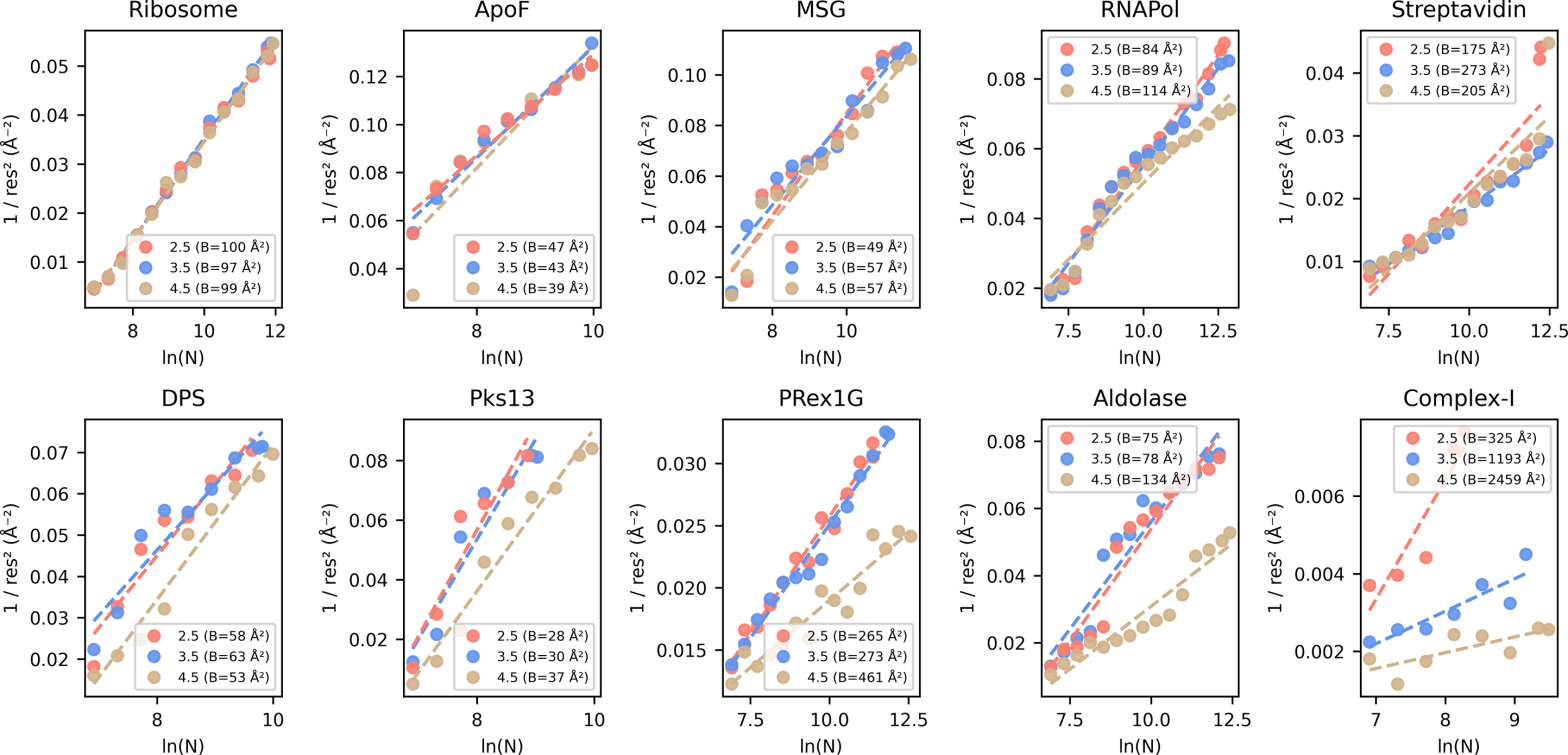
ResLog analysis of the *CryoSift* thresholds. Validation of the reconstruction quality using the ResLog plot (inverse resolution over the logarithm of the number of particles). The slope of the linear regression line, given as the *B* factor in Å^2^, is an indicator of the data quality (with lower meaning worse quality). Plots were calculated using *cryoSPARC*’s ResLog analysis at FSC 0.134. Half-set splits were generated from *ab initio* reconstructions using the ‘Homogeneous reconstruction only’ job to preserve the initial angular assignments.

Degrading resolution and reduction in map interpretability upon the removal of particles with stricter labels (cutoff 2.5) is an established observation and correlates the number of particles with a maximum resolution reachable, termed ResLog analysis (Stagg *et al.*, 2014 ▸). Using the *cryoSPARC* implementation, we calculated the ResLog slope, which is related to the *B* factor, for each selected 2D assessor threshold using the ResLog analysis (Fig. 5 ▸).

The strongest improvement in map interpretability (*Q*-score) and overall resolution (FSC 0.5) is also visible as increases in the ResLog slope and thus increased particle quality when comparing the 3.5 with the 4.5 *CryoSift* cutoffs for Complex-I, aldolase and PRex-1G. DPS, Pks13 and RNAPol also show improvements that match our earlier observations (Fig. 4 ▸). Almost no changes are visible for the ribosome and ApoF data sets, since they retain most of their particles when applying the different cutoffs (Fig. 3 ▸*b*).

To test the cross-platform usability of *CryoSift*, we transferred pre-processed particle stacks of Pks13, DPS and Complex-I from *cryoSPARC* to *RELION*5 using *pyem* and generated new 2D class averages using *RELION*’s 2D classification (*VDAM*). Subsequent class labeling with our *CryoSift* server demonstrates its ability to work on both *RELION* and *cryoSPARC* data (Supplementary Fig. S4).

Direct comparison of *RELION*5-processed classes using its *Class Ranker* and *CryoSift* using quality-sorted classes demonstrates a quasi-linear correlation across the two class labelers. While the Complex-I data set shows linear agreement across the entire labeling range, Pks13 and DPS show variations in assigned class quality for mid-tier class qualities, indicating a finer-grained quality estimation of *CryoSift* over *RELION*’s *Class Ranker* (Supplementary Fig. S5). This observation, consistent with the validation of the CNN (Fig. 1 ▸*c*), potentially points to diverging class-quality assignment by the curators during training.

## 4. Discussion

In this paper, we present a platform-agnostic 2D class-average assessor, termed *CryoSift*, that addresses a key bottleneck in cryo-EM data processing: unsupervised automation across user and experience levels with minimal integration efforts. We demonstrate the versatility of *CryoSift* by integrating it into *cryoSPARC* using the *cryosparc-tools* API, creating a fully automated workflow that accommodates users across different experience levels. At its core, the CNN-based *CryoSift* labels 2D class averages with quality scores, which can be used either as a standalone application for assessing *RELION* or *cryoSPARC* 2D averages, or as an integrated component within automated processing pipelines (such as *cryoSPARC*’s ‘Select 2D’ job utilizing the *cryosparc-tools* API). This iterative approach to 2D classification, quality labeling and selection helps to promote the inclusion of rare views that would otherwise be discarded in non-iterative procedures (Vilas *et al.*, 2022 ▸). A systematic evaluation of ten diverse data sets using FSC-based resolution estimates, *Q*-score atom-inclusion metrics and *B*-factor analysis revealed that a quality-score cutoff of 3.5 provides an optimal balance between including rare views (lower quality classes) and excluding unsuitable classes or false-positive picks.

While symmetric, high-molecular-weight targets (such as ApoF and DPS) generate stable 2D classes without meaningful improvements from iterative classification (Figs. 3 ▸*b*, 4 ▸*e*, 5 ▸ and Supplementary Fig. S6), most research-relevant samples suffer from conformational heterogeneity, compositional variability and preferred orientation effects. Our aldolase data set exemplifies how iterative classification successfully removes particles affected by partial denaturation and localized damage, as observed in EMDB entry EMD-21492, demonstrating the value of the tool for real-world applications (Fig. 4 ▸*e*, Supplementary Fig. S6).

A notable strength of this pipeline is its multi-tiered accessibility. Users new to cryo-EM analyses can upload data to our web server to familiarize themselves with expert-trained quality assessments, then apply default *cryosparc-tools* settings to their own data sets. Intermediate-level users can adjust parameters for their specific targets and implement parallel processing workflows. Advanced users and developers can contribute to CNN optimization by sharing labeled data sets, extend workflows to more challenging systems (for example filaments and membrane proteins) and combine *cryosparc-tools* with platform-conversion utilities for cross-platform automation. We have demonstrated this using *pyem* for file conversion of *cryoSPARC* particle stacks to *RELION*, and performed a direct comparison using three different data sets, which highlight easy cross-platform use of *CryoSift* with comparable class-quality labeling (Supplementary Fig. S4). While this manual approach of class-quality labeling using *CryoSift* for *RELION* data can be informative, the greatest benefit arises from automation using inbuilt cutoffs and iterative 2D classification with pooling of particles across multiple 2D classification and selection jobs. Consequently, developing a *RELION*-compatible iterative workflow would result in higher reconstruction quality and reduces the need for user intervention. In its current state, a conversion and transfer to *cryoSPARC* with subsequent use of our *CryoSift* and *cryosparc-tools* pipeline would enable easy automation of 2D class selection.

We envision *CryoSift* to serve as a community-driven tool that improves through user contributions. The web server enables users to contribute their own class labels, allowing continuous model refinement with diverse sample types and conditions. This crowdsourced approach has the potential to make the model increasingly accurate and generalizable over time. Further, this tool has relevance in educational purposes, as it provides clear examples of quality differences in 2D class averages, making it valuable for cryo-EM training programs.

We note that several extensions would further enhance the utility of *CryoSift*. The substantial fraction of false-positive picks observed in our data sets (Fig. 3 ▸*b*) suggests that automatic clustering of picked particles (*cryoSPARC* v.4.6+) without requiring user-defined inputs could increase the processing speed and improve reconstructions. Beyond quality-based selection, incorporating projection-level information to assess compositional heterogeneity, detect preferred orientation effects and compare particle distributions with simulated projections would provide valuable additional capabilities. Further, our assessment tool currently focuses on overall class quality rather than distinguishing between different conformers, oligomeric states or contaminants. While this approach effectively removes poor-quality classes, future development could incorporate more sophisticated classification schemes.

Our integration of *CryoSift* into *cryoSPARC* and planned incorporation into the *Magellon* platform for cryo-EM visualization, management and processing (Khoshbin *et al.*, 2025 ▸; https://www.magellon.org/) represents a step towards fully automated cryo-EM processing pipelines that maintain user control while reducing the burden of repetitive tasks. We anticipate that this approach has the capacity to accelerate structure determination and increase the accessibility of high-quality cryo-EM analyses to the broader scientific community.

## Supplementary Material

EMDB reference: mouse apoferritin, EMD-70880

Supplementary Figures. DOI: 10.1107/S2053230X25008866/ih5009sup1.pdf

Video interview with the authors. DOI: 10.1107/S2053230X25008866/ih5009sup2.mp4

## Figures and Tables

**Table 1:** Publicly available data sets used to train *CryoSift* Data sets marked with an asterisk were also used for the mass estimator.

Target	EMPIAR	EMDB	Target	EMPIAR	EMDB	Target	EMPIAR	EMDB
E3 Ub ligase	EMPIAR-11501	EMD-16087	Pol-SN	EMPIAR-11522	EMD-28786	Urease	EMPIAR-10389	EMD-10835*
Nucleosome	EMPIAR-11840	EMD-16859*	Primase	EMPIAR-11131	EMD-26346	bGal	EMPIAR-10061	EMD-2984*
E3 Ub ligase	EMPIAR-11734	EMD-41996	PreP	EMPIAR-10937	EMD-22278	RNApol	EMPIAR-10709	EMD-12885*
Antibody	EMPIAR-11341	EMD-28537*	ALK	EMPIAR-10930	EMD-24095	PA3488	EMPIAR-11204	EMD-32438*
AP2 Tgn38	EMPIAR-11605	EMD-24712*	CST	EMPIAR-10718	EMD-21567	GS-GN	EMPIAR-11139	EMD-14587*
AP2 heparin	EMPIAR-11604	EMD-24711	RNAPol-III	EMPIAR-10168	EMD-4180	FAsyn	EMPIAR-10470	EMD-4578*
Pks13	EMPIAR-11608	EMD-26574	RAG1-2	EMPIAR-10049	EMD-6487	Cas7-11	EMPIAR-11268	EMD-14848*
MSG	EMPIAR-11167	EMD-34029	N2ase	EMPIAR-11795	EMD-26764*	T20S	EMPIAR-10025	EMD-6287*
Aldolase	EMPIAR-10379	EMD-21492*	PRex-1G	EMPIAR-10285	EMD-20308*	CDK	EMPIAR-10561	EMD-12042
MAVS	EMPIAR-10031	EMD-6428	HA-trimer	EMPIAR-10097	EMD-8731	7TM-1G	EMPIAR-10288	EMD-0339

**Table 2:** Laboratory data sets used for training *CryoSift*

Target	Comment	Target	Comment	Target	Comment
Tubulin	Heterodimer	RNA	80 kDa	Kinesin binder	Complex
Protein	70 kDa	RNase P	RNA-free	HIV-1	Hexamer
Spike	SARS-CoV2	GPCR	Complex	40S	Ribosome
Aldolase	EMD-43528	PolQ	Polymerase	PolQHel	Polymerase
PolQHelNt	Polymerase	LONP1	Protease	NPM1	Pentamer
CRBN-DDB1	Ligase	TTR	Tetramer		

**Table 3:** Test data-set parameters for 2D class-average assessment in *CryoSift*

Target	EMPIAR	PDB code	Images used	Voltage (keV)	Pixel size (Å per pixel)	Particle diameter (Å)
Aldolase	EMPIAR-10379	6ald	1118	200	0.91	100
DPS	EMPIAR-11792	6gcm	194	300	0.834	100
Streptavidin	EMPIAR-10641	7dy0	2273	300	0.40	60
70S ribosome	EMPIAR-11524	8buu	6384	300	0.82	270
P-Rex1G	EMPIAR-10285	6pcv	1734	300	1.0	70
ApoF	EMPIAR-12798	7vd8	277	200	0.74	130
RNAPol	EMPIAR-11522	8f1k	6088	300	1.083	190
MSG	EMPIAR-11167	7yqm	700	300	0.822	90
Pks13	EMPIAR-11608	8cuz	100	300	0.835	170
Complex-I	EMPIAR-11656	7b93	571	300	1.055	290

## Data Availability

The in-house mouse apoferritin data set used for testing was deposited as EMD-70880, and aligned dose-weighted micrographs are available from the Electron Microscopy Public Imaging Archive (EMPIAR-12798). *CryoSift* is available on GitHub (https://github.com/sstagg/Magellon/tree/main/Sandbox/particle_processor) and the *CryoSift *web server is available at https://www.cryosift.org.
